# The Complete Genome Sequence of *n*-Alkane-Degrading Desulfoglaeba alkanexedens ALDC Reveals Multiple Alkylsuccinate Synthase Gene Clusters

**DOI:** 10.1128/MRA.00119-20

**Published:** 2020-04-23

**Authors:** Christopher R. Marks, Amy V. Callaghan, Irene A. Davidova, Kathleen E. Duncan, Brandon E. L. Morris, Michael J. McInerney, Joseph M. Suflita

**Affiliations:** a Department of Microbiology and Plant Biology, University of Oklahoma, Norman, Oklahoma, USA; b Biocorrosion Center, University of Oklahoma, Norman, Oklahoma, USA; Georgia Institute of Technology

## Abstract

Anaerobic alkane metabolism is critical in multiple environmental and industrial sectors, including environmental remediation, energy production, refined fuel stability, and biocorrosion. Here, we report the complete gap-closed genome sequence for a model *n*-alkane-degrading anaerobe, Desulfoglaeba alkanexedens ALDC.

## ANNOUNCEMENT

Despite advances in the study of anaerobic hydrocarbon biodegradation, there is a dearth of sequenced genomes from representative isolates (1). Desulfoglaeba alkanexedens strain ALDC was obtained from a U.S. Navy oil wastewater storage facility (2). The organism can completely oxidize C_6_ to C_12_ *n*-alkanes to CO_2_ coupled to sulfate reduction or syntrophic methane production with initial substrate activation via the fumarate addition pathway (2–4). Early sequencing and proteomic efforts helped focus attention on the prospect for several alkylsuccinate synthase gene clusters and multiple nucleotide repeats. It was decided that longer reads were needed to close the genome. To that end, D. alkanexedens was cultivated for genomic DNA extraction at 31°C in a reduced basal marine medium supplemented with yeast extract and butyrate (5). Biomass was harvested from an exponential-phase culture by centrifugation in sealed polycarbonate vessels. The resulting cell pellet was resuspended in Qiagen genomic tip bacterial lysis buffer B1 modified with a 10-fold-higher concentration of EDTA (46.5 g liter^−1^ Na_2_EDTA). Genomic DNA was extracted using the genomic tip DNA extraction kit (Qiagen, Inc., Valencia, CA) as described by the manufacturer. Libraries were prepared for Pacific Biosciences single-molecule real-time (SMRT) sequencing following the protocol for 20-kb template preparation using a BluePippin size selection system (https://www.pacb.com/wp-content/uploads/2015/09/Procedure-Checklist-20-kb-Template-Preparation-Using-BluePippin-Size-Selection.pdf) using a template preparation kit v1.0. The size-selected SMRTbell library (BluePippin cutoff value, 10 kb) was sequenced on the RS II platform using standard MagBead loading, P6-C4 chemistry, and 6-h data collection times. Postfiltering quality control analysis confirmed low adapter dimer levels and adequate read length, quality, and quantity to proceed with genome assembly using the HGAP2 algorithm, which was performed by the Washington State University Molecular Biology and Genomics Core Facility. The genome sequence of the single resulting contig was uploaded to the JGI Integrated Microbial Genomes (IMG) v4 pipeline (GOLD Analysis Project accession number Ga0097685) for automated annotation and public access (6). All downstream phylogenetic analyses and refined sequence alignments were conducted using the MEGA6 software package (7). Manual annotation of open reading frames associated with alkane metabolism was performed using the DNASTAR software suite (DNASTAR, Madison, WI).

The genome of D. alkanexedens ALDC is a single circular chromosome, 3,365,583 bp long, with an average molar G+C content of 59.31%. A total of 3,020 protein-coding genes were identified, and 2,270 of those were assigned a predicted function by the IMG annotation pipeline. The genome contains 62 RNA genes, specifically 6 rRNA genes (2 copies each of 5S, 16S, and 23S rRNA genes), 47 tRNA genes, and 9 miscellaneous categorized RNA genes. The 16S rRNA genes are 99.9% similar to each other and are both located on the minus strand at locus tags 111218 and 113015.

Two distinct gene clusters encoding the requisite subunits for alkylsuccinate synthases were identified. Gene cluster 1 is on the minus strand and contains the requisite genes *assABCDE* and *masE*, as well as ancillary genes for large and small subunits of methylmalonyl-coenzyme A (CoA) mutase, an ATP-dependent lysine-arginine-ornithine (LAO)/arginine-ornithine (AO) transport system, and an MmgE/PrpD family protein. Gene cluster 2 is on the plus strand and contains genes functionally homologous to those in cluster 1 but also contains *assF* and a gene coding for a putative carboxyl transferase. The *assA1* and *assA2* sequences were highly similar to each other, with a maximum amino acid sequence dissimilarity of 3.1%. AssA homologues were most closely associated with AssA1 (Dalk_1731) of strain AK-01, with sequence identity of 77% and residue chemistry similarity of 88%. Alignment of each AssA protein sequence revealed the proposed catalytic glycine residue and single cysteine residue (distinct from the conserved tandem cysteine for pyruvate-formate lyases) conserved among known AssA homologues (8). Further research is required to evaluate the potential for differential expression of the two gene clusters.

### Data availability.

This genome is available from the NCBI GenBank database under BioProject accession number PRJNA541298 and GenBank accession number CP040098. Unassembled sequence reads are available under the accession number SRX7796451.
